# Establishment of a regenerative endodontic procedures model of mature mouse teeth and evaluation of the wound healing process

**DOI:** 10.1007/s10266-025-01211-4

**Published:** 2025-09-29

**Authors:** Xiuting Wang, Shigeki Suzuki, Shin-Ho Tsai, Karin Nagasaki, Rahmad Rifqi Fahreza, Masato Omori, Satoru Yamada

**Affiliations:** 1 Department of Periodontology and Endodontology, Tohoku University Graduate School of Dentistry, Sendai, Japan; 2 Department of Operative Dentistry, Okayama University Graduate School, Medicine, Dentistry and Pharmaceutical Sciences, 2-5-1 Shikata-cho, Kita-ku, Okayama, 700-8525 Japan

**Keywords:** Regenerative endodontic procedures, Establishment of protocols, Mouse experimental model, Mature teeth

## Abstract

As the pulp regeneration for non-vital teeth is one of the ultimate clinical achievements, regenerative endodontic procedures (REPs) have become the most explored treatment modality. In this technique, periodontal tissue is guided from the apical region into the root canal and pulp chamber to promote attachment. It is well established that immature teeth are effective targets for treatment. However, the indications for this treatment have not yet expanded sufficiently to encompass mature teeth with closed apical apex. In the present study, a mouse model of REPs in mature teeth was established, employing the maxillary first molar mesial root. μCT analyses disclosed that the distance from the occlusal surface to the physiological apex of the maxillary first molar mesial root in mice is 2.14 mm ± 0.08 mm, and the distance from the occlusal surface to the periapical alveolar bone is 2.46 mm ± 0.10 mm. Mesial root canal was treated with several sizes of k-files, and 15# k-file was identified as the most suitable k-file for use (*P* = 0.0007). During the regenerative process, spindle-shaped fibroblast-like cells, fibrous tissue formation, and mineralized tissue formation were identified on days 14 and 28. This study demonstrated that it is feasible to use the maxillary first molar mesial root as a REPs model for mature teeth and provided a detailed protocol and analysis of the healing process.

**Supplementary Information:**

The online version contains supplementary material available at 10.1007/s10266-025-01211-4.

## Introduction

Dental pulp, a connective tissue, is enveloped by dentin and serves multiple functions, including dentin formation, immune protection, and nutrient supply [1]. The prevailing approach among clinical dentists involves the utilization of traditional root canal filling following a meticulous process of disinfection of the root canal. However, it should be noted that certain disadvantages persist, including the potential for tooth fracture and re-infection due to the loss of vital pulp tissue in the tooth. Nygaard-Østby initially proposed a methodology that involved the filling of a blood clot within the root canal following the removal of the entire pulp during the 1960s [2]. The aforementioned elements thus constitute the foundational principles of regenerative endodontics. A comparison of regenerative endodontic procedures (REPs), a type of cell-homing strategy, with cell transplantation therapies reveals several notable advantages. These include the promotion of apical lesion healing, the mitigation of symptoms, and the facilitation of continuous root development [3, 4]. Consequently, it is regarded as the fundamental objective of endodontic therapy.

In recent years, researchers have conducted clinical and animal experimental research on REPs [5, 6]. The clinical operation was performed in accordance with the recommendation established by the American Association of Endodontists [7]. This recommendation entailed the occurrence of bleeding into the canal system as a consequence of over-instrumenting. Anastasia et al. reported the successful observation of the healing of the lesion accompanied by the closure of the root canal in immature premolar pulpal necrosis with a chronic apical abscess after utilizing this regenerative procedure [5]. In addition, large animals have been utilized in REPs with the observation regarding the presence of vascularized tissue and dentinal formation [6]. Even though rodent teeth are tiny and not commonly used in REPs [8], some researchers have conducted REPs studies in rats and found cementum tissue and connective tissue formation [9, 10]. The significant advantages of clinics, including their simplicity and the preservation of vital tissue, suggest that the REPs offer promising application prospects [11, 12].

Despite the substantial body of research on REPs, there is a clear need for further refinement and improvement in this field in the future. Initially, the predominant clinical practice among dental dentists involved the selection of immature teeth for REPs. However, there is a growing recognition of the importance of incorporating pulp-ectomized mature teeth in REPs as well [13]. Second, it is evident that the tissues formed in the pulp chamber by REPs include cementum/bone–periodontal ligament complex-like tissue [14]. A molecular biological exploration of REPs is imperative for the regeneration of pulp tissue by physiological dentin-pulp complexes. Therefore, it is important to note that the restoration of vascularity in the pulp tissue alone is insufficient; the functional regeneration of the dentin–pulp complex is also necessary in the field of regenerative endodontics [15]. Besides, infection control also should be emphasized in order to achieve functional regeneration. To reduce the negative impact of residual bacteria on REPs, such as continued stimulation of inflammation and impact on remaining dentin development, researchers have tried many intracanal sterilization medicines, such as triple antibiotic pastes [16] and Azithromycin-laden chitosan hydrogel [17]. When performing REPs, it is recommended to use NaOCl at a low concentration that is not harmful to undifferentiated mesenchymal cells present in the apical periodontal tissue [17]. In particular, immature teeth require root canal cleaning that does not damage the dental papilla. Consequently, the mechanism of REPs-induced real pulp–dentin complex regeneration is of vital importance to be discovered. To further investigate the mechanisms underlying regeneration, the use of genetically modified animals is anticipated.

In this study, a mature mouse REPs model was established to investigate the regeneration process. The objective of this study is to establish a foundation for the subsequent analysis of the regenerative mechanisms involved.

## Materials and methods

### Animals

All experimental procedures were conducted in accordance with the “Regulations for Animal Experiments and Related Activities at Tohoku University.” Prior to the initiation of these procedures, they were reviewed by the Institutional Laboratory Animal Care and Use Committee of Tohoku University and approved by the President of the University (Permit No. 2021DnA-014-08). The experimental model consisted of 9-week-old C57BL/6J male mice. All mice were purchased from CLEA Japan, Inc. (Osaka, Japan) and randomly allocated in each experiment. The mice were provided with a standard rodent diet and water. All the surgical procedures were applied by a single calibrated and experienced operator using a light microscope (Leica S Apo Stereozoom, Leica, Wetzlar, Germany).

### μCT analysis

μCT was conducted as previously described [18]. In summary, fixed maxillae were scanned with a μCT scanner (Scanxmate-E90, Comscantecno Co., Ltd., Yokohama, Japan) and recorded at 70 kV/114 μA using Xsy FP software (version 2.1, Comscantecno Co., Ltd.,). The 3D images were reconstructed using coneCTexpressI software and TRI/3D-BON software for μCT analysis.

### Measure the distances from the occlusal surface to the physiological apex and the periapical alveolar bone of the mesial root of the maxillary first molar

**Fig. 1:**
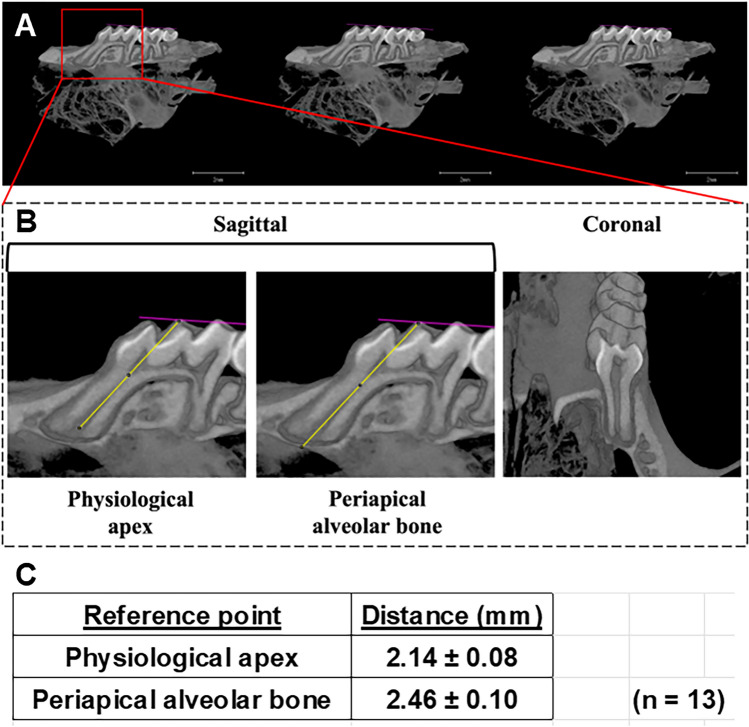
Measurement of the distances from the occlusal surface to the physiological apex and to the periapical alveolar bone of the mesial roots of the maxillary first molar. **A**, **B** Sagittal and coronal μCT images of the teeth. The distances from the occlusal surface to the physiological apex and to the periapical alveolar bone of the right maxillary first molar are shown as yellow lines. **C** The average distances from the occlusal surface to the physiological apex of the maxillary first molar mesial root and the distance from the occlusal surface to the periapical alveolar bone

The distances from the occlusal surface to the physiological apex and to the periapical alveolar bone of the maxillary first molar mesial root were determined by μCT analysis. The method of Elisheva G. was adopted [19]. The images of each mesial root canal of the right first maxillary molars without surgery were taken under the condition of 70 kV, 114 μA, and 12.902 μm/pixel. Among these mesial root canal images of each mouse, 3 images reflecting the apical constriction were selected, and the occlusal surface was indicated by drawing a pink line (Fig. 1A). Then, the distances from the occlusal surface to the physiological apex and to the periapical alveolar bone of the mesial root were measured by Image J (Fig. 1B, yellow lines).

### Selecting an appropriate k-file for a regenerative endodontic procedure

Nine-week-old mice were gently anesthetized intraperitoneally, the left maxillary surgical site of the mouse was sterilized with 75% ethanol, and the enamel and dentin in the crown of the left maxillary first molar were removed with a 1/4# round bur (Morita, Osaka, Japan). Then, the speed of rotation was reduced when the pulp chamber was exposed. The remaining crown pulp was removed using a micro-excavator (New O.K. Micro Exca, Setoseisakusyo, Ibaraki, Japan) without destroying the pulp chamber floor and surrounding dentinal crown walls. After 3 root canal orifices of the left maxillary first molar were clearly visible, the sterilized cotton was used to dry the pulp chamber. The root canal orifices of the distal root and the palatal root were directly capped with composite resin (Beautiful Flow Plus F100, Shofu, Kyoto, Japan) and the resin was hardened with a light-curing machine. A 6# k-file was inserted into the mesial root canal to remove pulp tissue (6# k-file group). 6# and 8# k-files were inserted to remove pulp tissue (8# k-file group). Similarly, the medial root canals were treated with 6#, 8#, and #10 k-files in sequence (10# k-file group) and were treated with 6#, 8#, #10, and #15 k-files in sequence (15# k-file group). Next, these mice were euthanized immediately after surgery, and preparation of tooth sections was performed. To compare the removal efficiency of pulp tissue between groups, the entire root canal space and the remaining pulp tissue area in the mesial root canal were calculated from H&E-stained section images using Image J. Four serial sections in the middle of the mesial root canal from each tooth sample were used for statistical analysis. During the procedure, the operator not only confirmed the apex using their fingertips but also proceeded with the treatment while confirming the working length using a caliper (Supplemental Fig. 1).

### Regenerative endodontic procedures (REPs)

The subjects were divided into three groups: control group, REPs group, REPs without irrigation group. In the REPs group, the pulp chambers of 9-week-old mice were exposed and the root canal orifices of distal root and palatal root were directly capped with composite resin, and the resin was hardened with a light-curing machine as described above. Then, 6# k-file, 8# k-file, 10# k-file, and 15# k-file (21 mm, Mani, Japan) were inserted into the mesial root canal of the left maxillary first molar to reach the apical constriction. Following the enlargement of the mesial root canal with a 15# K-file, the canal was irrigated with 50 μL of solution containing 1.5–3% sodium hypochlorite (NaOCl) (Dental Antiformin, Nishika, Yamaguchi, Japan) and 0.9% saline (Biosciences, CA, USA). Subsequently, 50 μL of 17% ethylenediaminetetraacetic acid was introduced into the canal for a duration of 5 min. Sterilized paper points (15#, Meta Biomed, Chungcheongbuk-do, Korea) were utilized to remove the residual irrigant and dry the interior of the root canals. The REPs without irrigation group were treated similarly but without irrigation steps. Next, a new pre-curved 15# k-file was inserted and penetrated the apical constriction to induce bleeding from the apical periodontal tissue into the canal. After 5 min of bleeding, the level of the cementoenamel junction was confirmed. Then, the mesial root orifice and the pulp chamber were directly capped with composite resin, and the resin was hardened with a light-curing machine to seal completely. In the control group, the mesial root of the left maxillary first molar of the mice was only pulp-ectomized, and then the mesial root orifice and the pulp chamber were capped as same as REPs groups. A simple moisture barrier was created by placing sterilized cotton on the buccal side, and all procedures were performed. Some representative images during the surgery were exhibited in Supplemental Fig. 3. The mice were then maintained for 0, 3, 14, and 28 days. The flow diagram of this study was described in Supplemental Fig. 2. The mice were perfused with DPBS and 4% paraformaldehyde. Then, the maxillae were dissected and kept in 4% paraformaldehyde for 24 h at 4 °C.

### Histological analysis

The fixed maxillae were utilized for hematoxylin and eosin (H&E) staining and Masson's trichrome (MT) staining following μCT scanning. The decalcification process was carried out using 0.134 mol of ethylenediaminetetraacetic acid at a temperature of 4 °C for a period of 14 days. Following this, the samples underwent a series of dehydration steps, involving the gradual replacement of water with increasing concentrations of ethanol. Subsequently, the samples were transferred into xylene and then embedded in paraffin. Staining was performed on 5-µm-thick paraffin sections. Histological images were captured using an upright microscope (DM6000 B: Leica, Wetzlar, Germany) with a digital camera (DP28: Olympus, Tokyo, Japan). The histopathological parameters were measured as previously described [6, 10, 20, 21]. Briefly, as shown in Table 2, using ImageJ software, a straight line along the long axis of the mesial root, connecting the pulpal chamber floor to the root apex, was drawn in the histological images obtained from mesio-distal sections. Then, based on this line, the mesial root was divided into three parts having equal distance: the cervical, the middle, and the apical, following the direction from the pulpal chamber floor to the root apex. The scores for different types of tissue—fibrous tissue, mineralized tissue, and immature granulation tissue—were evaluated based on specific histopathological parameters. For fibrous and mineralized tissues, the scoring criteria were as follows: a score of 0 points indicated no new tissue generation in the root canal, a score of 1 point was given when new tissue was primarily deposited at the root apex, a score of 2 points was assigned when tissue was deposited at both the root apex and the middle section, and a score of 3 points indicated that new tissue was distributed throughout the entire root canal. For immature granulation tissue, a score of 0 points was assigned if no tissue was generated in the root canal, while a score of 1 point was given if immature granulation tissue was present.

### Exclusion criteria

Exclusion criteria are listed in Table 1.

**Table 1:** Exclusion criteria

Intraoperative exclusion criteria
1. Saliva contamination
2. Bleeding before destruction of apical foramen
3. Incomplete resin sealing of other two orifices

Postoperative exclusion criteria
1. Unhealthy mice
2. Filling resin detachment
3. Root canal perforation
4. Damaged apical foramen in control group

### Statistical analysis

**Fig. 2:**
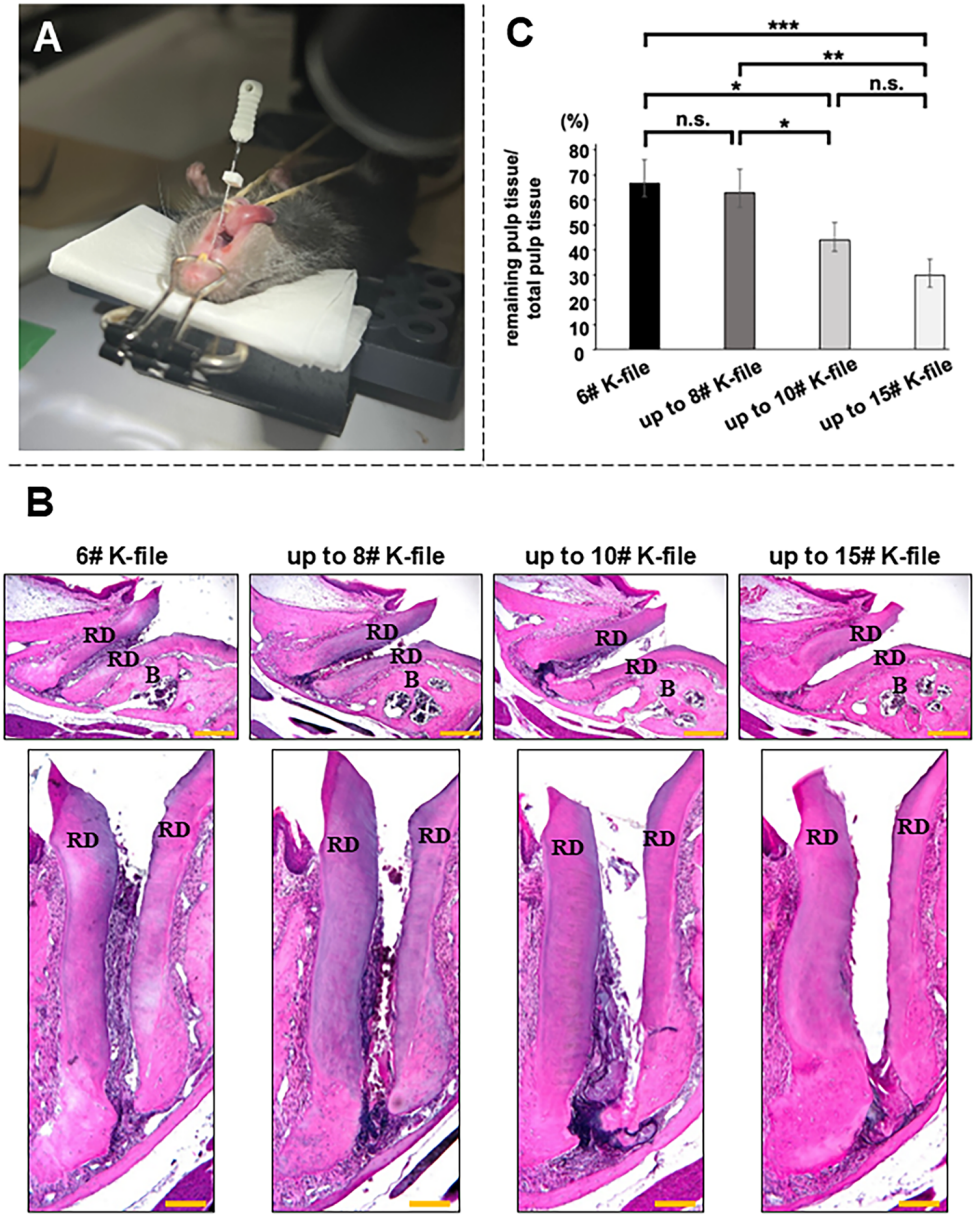
Quantification of residual pulp tissue in root canals enlarged to four different sizes. **A** An actual surgical operation. **B**, **C** Pulp chambers were exposed and enlarged with 6# k-file (6# k-file), with up to 8# k-files (up to 8# k-file), with up to #10 k-file (up to #10 k-file), with up to #15 k-file (up to #15 k-file). Demineralized male maxillary sections collected shortly after surgery and stained with H&E. Scale bars correspond to 500 and 200 μm at low and high magnification, respectively. Residual pulp tissue/total pulp tissue was calculated (*n* = 3). *p < 0.05; **p < 0.01; ***p < 0.001 significantly different. *n.s.* not significant, *RD* root dentin, *B* alveolar bone

Statistical analysis was performed by one-way ANOVA, followed by Tukey’s test, and* p* < 0.05 was considered significant (Fig. 2). Mann–Whitney *U* test was used to analyze results in Table 2 and *p* < 0.05 was considered significant.

**Table 2:** Histologic observations of the healing process at 28 days (with or without irrigation)

Scores for each parameter	Experimental groups (*n* = 8)		*p*-value
	REPs group	REPs without irrigation group	
Fibrous tissue formation			
0 = Absent	0/8	0/8	0.045*
1 = up to apical	4/8	1/8	
2 = up to middle	4/8	4/8	
3 = up to cervical	0/8	3/8	
Mineralized tissue formation			
0 = Absent	2/8	1/8	0.292
1 = up to apical	5/8	4/8	
2 = up to middle	1/8	3/8	
3 = up to cervical	0/8	0/8	
Granulation tissue formation			
0 = Absent	0/8	3/8	0.075
1 = Presence	8/8	5/8	

*REP* regenerative endodontic procedure

*The presence of a significant difference between the groups (*p* < 0.05). The data were analysed using the Mann–Whitney *U* test

## Results

### The distances from the occlusal surface to the physiological apex and from the occlusal surface to the periapical alveolar bone of the mesial root of the maxillary first molar

Utilizing images of the sagittal plane obtained by µCT, the distances from the occlusal surface to the physiological apex and periapical alveolar bone were measured (Fig. 1A, B). Statistical analysis indicated that the distances from the occlusal surface to the physiological apex and to the periapical alveolar bone of the maxillary first molar mesial root in mice were 2.14 mm ± 0.08 mm and 2.46 mm ± 0.10 mm on average, respectively (Fig. 1C).

### The least amount of pulp tissue remaining in the root canal with a 15# k-file application

The experimental scene shows the exposure of the pulp of a fixed mouse tooth and the insertion of a k-file into the mesial root canal of the left maxillary first molar (Fig. 2A). H&E staining revealed that all sizes of k-file successfully entered the mesial root canal of the left maxillary first molar and reached the apical constriction. However, because the procedure failed to enlarge the root canal sufficiently up to the enlarged 6# k-file, 8# k-file, and 10# k-file sizes, a lot of residual pulp tissue was identified within the root canal (Fig. 2B). On the other hand, the procedure up to the enlarged 15# k-file showed excellent ability to thoroughly remove pulp tissue and widen the root canal space (Fig. 2B). Quantification analysis showed that the 15# k-file enlargement technique removed the maximum amount of pulp tissue (Fig. 2C). Therefore, the 15# k-file was used in the following experiment.

### Histological observations of the healing process of REPs

**Fig. 3:**
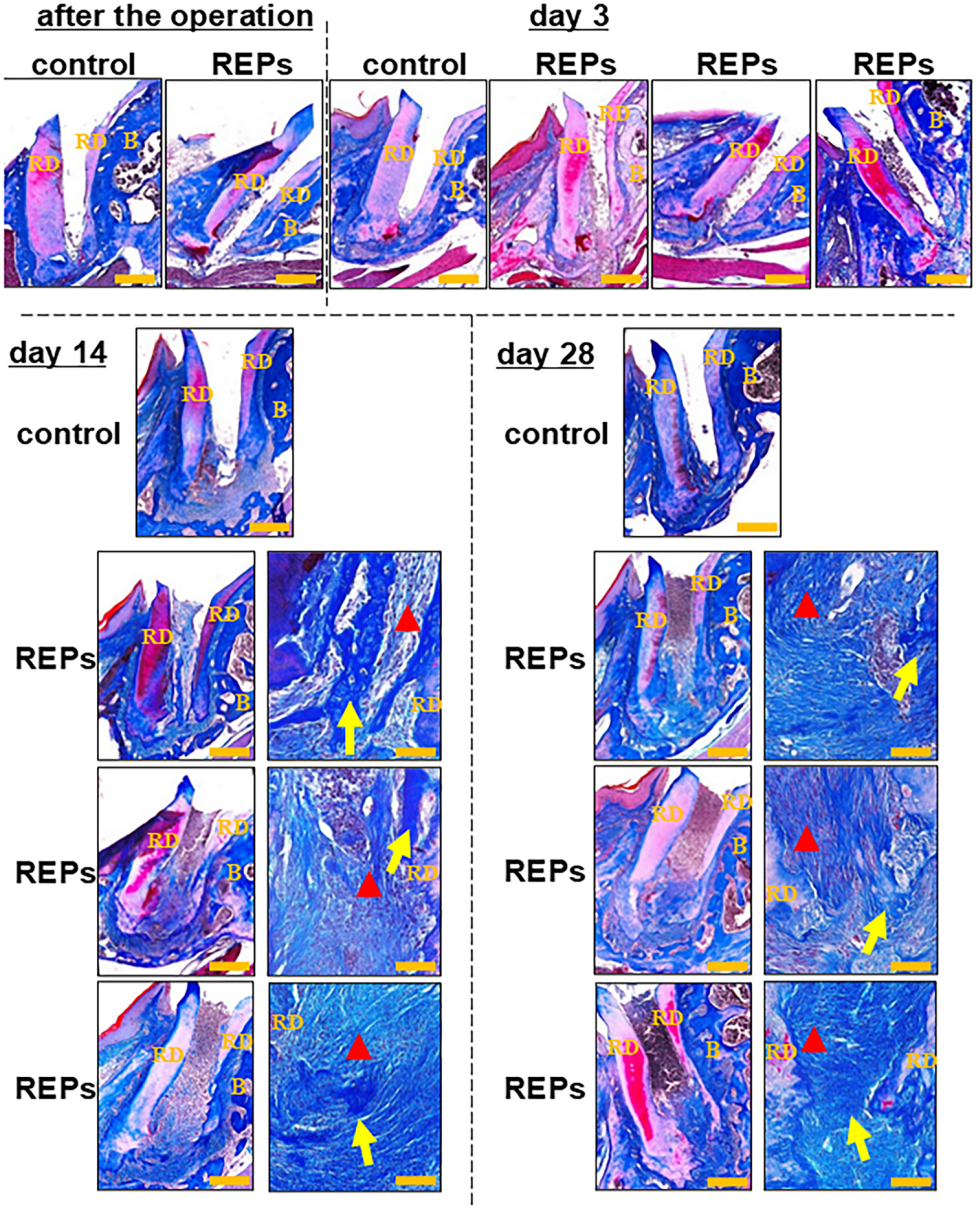
Histologic observations of the postoperative regenerative process. Demineralized male maxillary sections taken shortly after surgery and on days 3, 14, and 28 were stained with Masson’s trichrome (*n* ≧ 5). Scale bars correspond to 500 and 100 μm at low and high magnification, respectively. *REPs* regenerative endodontic procedures, *RD* root dentin, *B* alveolar bone. Red arrowheads indicate newly formed fibrous tissue. Yellow arrows indicate newly formed mineralized tissue

Pulp tissue was removed by the procedure up to the enlarged 15# k-file as described above, and the apical constriction was broken to induce bleeding from the apical periodontal tissue as described in the Materials and methods section (REPs group). Following the removal of the pulp tissue in the root canal, successful breaking of the apical constriction was identified in the REPs group (Fig. 3: after the operation). Although the blood clot disappeared in the root canal, presumably due to the perfusion process before collecting samples, the broken apical constriction and the remaining infiltrated erythrocyte components inside the root canal in the REPs group provided evidence of successful blood clot induction. On the other hand, in the control group, the root canal was enlarged as in the REPs group, but the apical constriction was not destroyed. Mice that met the exclusion criteria listed in Table 1 were excluded from the analyses. Three representative sections of the REPs group at day 3 showed the presence of blood cells within the root canal of the REPs group but not of the control group (Fig. 3: day 3). Furthermore, the presence of reticulated fibrin formation and blood cell infiltration in the apical periodontal tissue was also detected in the REPs group. Three representative sections of the REPs group at day 14 exhibited a significant presence of spindle-shaped fibroblast-like cells within the newly formed fibrous tissue (Fig. 3: day 14, red arrowhead), which was stained with light blue. It is noteworthy that newly formed mineralized tissues, which were stained with dark blue, were generated and localized from the apical to the middle of the root canal (Fig. 3: day 14, yellow arrow). The upper half of the root canals exhibited evidence of immature granulation tissue. Three representative sections of the REPs group on day 28 exhibited substantial infiltration of spindle-shaped fibroblast-like cells and induced fibrous tissue formation in the lower half of the root canals (Fig. 3: day 28, red arrowhead). Furthermore, the REPs group exhibited mineralized tissue formation (Fig. 3: day 28, yellow arrow). Conversely, the internal space of the root canal within the control group remained unoccupied on days 3, 14, and 28. These results indicated that the presence of regenerative tissue inside the root canal of the REPs group was attributable to the invasion of cells by REPs.

### Histologic observations of the healing process at 28 days after the regenerative endodontic procedures without the irrigation step

**Fig. 4:**
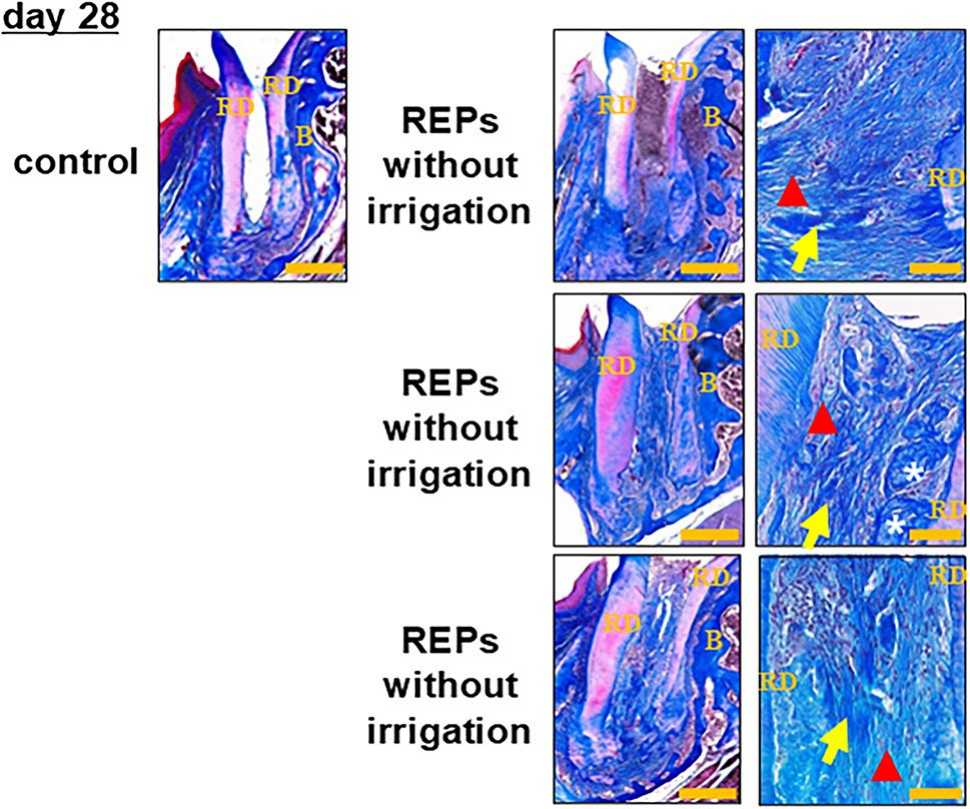
Histologic observation of the postoperative regenerative process after 28 days of REPs without irrigation. Demineralized male maxillary sections containing teeth that underwent regenerative endodontic procedures without an irrigation step were collected on day 28 and stained with Masson’s trichrome (*n* = 8). Scale bars correspond to 500 and 100 μm at low and high magnification, respectively. *REPs without irrigation* regenerative endodontic procedures without irrigation step, *RD* root dentin, *B* alveolar bone. Red arrowheads indicate newly formed fibrous tissue. Yellow arrows indicate newly formed mineralized tissue. White asterisks indicate dentin debris

The tissue regeneration process was clearly identified at days 14 and 28, but no drastic changes were observed between days 14 and 28 in the amount of connective tissue, the amount of hard tissue, or even the amount of immature granulation tissue. Therefore, to consider the possibility that the irrigation step prior to apical constriction breaking may reduce the regenerative capacity of REPs, REPs without the irrigation step were performed similarly to the teeth that underwent REPs, as shown in Fig. 3. As expected, the inside of the root canal of the control group remained empty at day 28 (Fig. 4). Three representative sections of the REPs without irrigation group at day 28 showed the drastic infiltration of spindle-shaped fibroblast-like cells and abundant fibrous tissue formation, which were clearly observed (Fig. 4: red arrowhead). Newly mineralized structures were detected, abundant in the REPs without irrigation group (Fig. 4: yellow arrow). The formation of immature granulation tissue, which is evidence of delayed tissue healing, was observed in all the samples of the REPs group, but it was only identified in 5 of 8 samples of the REPs without irrigation group on day 28 (Figs. 3 and 4, Table 2) Dentin debris remained inside the root canals due to the lack of irrigation step (Fig. 4: white asterisk).

## Discussion

REPs is one of the most promising methods of pulp–dentin complex regeneration [22]. Immature permanent teeth in children and adolescents have been focused on as the target population, but mature teeth are also included in the scope of research on REPs because vital pulp tissue regeneration revives the abilities of tooth immunity, restorative dentin formation, and color retention [23, 24]. Therefore, it is necessary to conduct studies on the application of REPs in mature teeth [25].

At present, the newly regenerated tissues by REPs are mainly cementum, periodontal ligament tissue, and bone, which means that REPs can promote the repair process but not the real regeneration of the pulp–dentin complex [15]. However, due to the ethical difficulties [11], clinical specimens have rarely been used for histological and molecular biological analyses to explore the key molecules of REPs that contribute to increasing the success rate and generating next-generation REPs that reconstruct the dentin-pulp complex rather than the cementum/bone–periodontal ligament complex. Most of the genetically modified animal models have been generated using mouse species; therefore, the development of the REPs model of mouse teeth is necessary. Based on this idea, the protocol using incomplete mouse roots was developed [26]. The significance of this study is the establishment of a protocol using mature mouse teeth. In this study, focusing on the mesial root of the maxillary first molar, the distance from the occlusal surface to the physiological apex was found to be 2.14 mm ± 0.08 mm (Fig. 1C). Enlarging the root canal to this working length means that the pulp tissue can be reliably removed without damaging the periodontal tissues outside the apical constriction. It was also found that the distance from the occlusal surface to the alveolar bone was 2.46 mm ± 0.10 mm. When guiding bleeding from outside the apical constriction into the root canal, setting the working length to 2.14–2.46 mm allows the procedure to be performed without penetrating the alveolar bone. In REPs, an adequate supply of cells and nutrients from the periodontal tissues is necessary for the regeneration of revascularized and reinnervated pulp tissue. However, it is also important to avoid excessive breaking of the apical constriction [27]. The results shown in Fig. 2 indicated that the 15# k-file was the best choice in the mesial root of the first upper molar of the mouse to remove the original pulp tissue and widen the space in the root canal for root canal irrigation and subsequent suction of the irrigants with paper points, and then to induce bleeding from the apex.

Successful pulp regeneration in the root canal was evaluated by 3 main parts: new angiogenesis, reparative mineralized tissue formation, and new connective tissue. First, all control groups reflected that the manipulation was sufficient to remove the original pulp tissue and odontoblasts (Figs. 3 and 4). In fact, it was unavoidable that a small amount of pulp tissue remained near the apex due to the original root canal structure of the mice with curvature surrounding the apical constriction, but the irrigation step with 1.5–3% NaOCl, 0.9% saline, and 17% EDTA could inactivate this tissue. Mineral trioxide aggregate (MTA) is widely used in regenerative endodontic procedures, which has the advantages consisting of tight sealing [28], antibacterial properties [29], and regulation of cell activity and mineralization [30]. One study suggests that infection in the upper region significantly affects root tip regeneration [31]. Therefore, the American Association of Endodontists recommends placing a resorbable matrix over the blood clot and then placing MTA as a capping material. In this study, the root canal orifice was sealed with resin instead of MTA. While MTA has several beneficial effects, this study did not find any significant problems with resin sealing. Therefore, at least in the mouse model, there may be no need to insist on the use of MTA. The tendency toward enhanced tissue repair was observed when the root canal was not irrigated on day 28 (Figs. 3 and 4, Table 2). This finding may be related to the irrigation process. To some extent, the irrigation process is considered to be a double-edged sword. The remaining irrigation solution in the root canal leads to a negative influence on cell behavior in the regeneration stage [32]. In spite of the appropriate paper points to adequately remove the irrigation solution, the small amount of irrigation solution may remain in the periapical area. Furthermore, the literatures suggest that the residual EDTA may compromise cell viability and disrupt cellular function and blood clot formation [32, 33]. Therefore, the side effect of residual irrigation solution should be considered in mouse REPs models. However, the present study concentrated on formulating manual techniques; consequently, experiments were conducted in root canals devoid of infection. Indeed, an analysis of the REPs model in a murine model with a history of infection suggests that root canal cleaning with NaOCl is essential; however, this procedure was not performed in the present study and will be considered in future research. It is evident from the findings of this study that irrigant residue is likely to occur in the mouse REPs model and has a significant impact. Moreover, due to the dimensions of the medullary cavity, it is practically impossible to employ double temporary filling with MTA and resin. A notable constraint of the mouse REPs model pertains to its inability to account for the tissue regeneration effects of MTA or other agents on cells/tissues guided into the root canal, a limitation that distinguishes it from more sophisticated models.

One study reported that by inducing bleeding in immature teeth in REPs, large numbers of mesenchymal stem cells were released from the periapical area and delivered into the root canal [34]. Although the apical papilla degenerated in mature teeth, Chrepa, et al. showed that enormous mesenchymal stem cells could also be infused into the root canal system by blood induction in REPs [35]. In this study, there was no apparent promotion of tissue regeneration from day 14 to day 28 (Fig. 3). The lack of promotion of tissue healing in the late healing phase and the failure to observe the formation of Masson-Trichrome-positive fibrous tissue up to the upper half of the root canal in the experimental group suggest that the activity and quantity of stem cells in the apical region of mature mouse teeth are not necessarily sufficient to reconstruct the entire root canal. In the present study, the regenerated tissue occurred during the long-term healing stage (Figs. 3 and 4). The fresh regenerated tissue consisted of abundant spindle-shaped fibroblast-like cell infiltration, connective tissue, and both immature and mature collagen fiber networks. Type I collagen is the most predominant component in the mineralized tissues, and more new collagen fiber formation probably indicates faster repair in the regeneration process [10]. Furthermore, the mineralized tissue with cellular components was also found to extend from the periapical area into part of the root canals (Fig. 3). Minic et al. made a detailed description of different types of achievement of tissue regeneration in their research and assumed that the cementum islands were formed via those migrating stem cells differentiating into cementoblast-like cells and depositing a matrix of collagen fibers; then the fibers calcify [36]. Mineralization during the regenerative phase is a complex process; different origins of stem cells can lead to distinct differentiation. Mesenchymal stem cells exist in the periapical region, such as periodontal ligament stem cells and alveolar bone mesenchymal stem cells [24, 37, 38]. Edanami et al. investigated different healing patterns after REPs based on the preoperative amount of remaining healthy pulp and apical tissues in immature rat molars and concluded that teeth with disorganization of pulp and apical tissues at 0.15–0.38 mm beyond the apex healed with periodontal ligament-like tissue, cementum-like dentin-associated mineralized tissue, and intracanal bone [39]. Some researchers suggested that the cementum-like tissue in the root canal was formed by periodontal ligament stem cells [40]. Further experiments to investigate the origin and characteristics of the newly formed cementum/bone-like tissue under the regenerative procedure are warranted to establish the new REPs methods that regulate the width and volume of hard tissue formation in the root canal and pulp chamber and induce the regeneration of the dentin–pulp complex rather than the cementum/bone–periodontal ligament complex.

In summary, it is concluded that maintaining the working length within the range of 2.14–2.46 mm can induce mature mouse REPs without causing damage to the surrounding periapical alveolar bone. Furthermore, the 15# k-file has demonstrated its efficacy in mice, yielding favorable REPs results. The long-term regeneration of mature mouse REPs can result in the formation of both fibrous and mineralized tissue. Furthermore, it is essential to take into account any residual irrigant during this process. In consideration of the findings from all experiments conducted, the REPs mouse model was successfully established with a high degree of precision. This development serves as a foundational framework for future research aimed at promoting dentin-pulp complex regeneration.

## Supplementary Information

Below is the link to the electronic supplementary material.

Supplementary file1 Supplemental Figure 1. Working length measurement with electronic liquid crystal caliper (TIF 213 KB)

Supplementary file2 Supplemental Figure 2. The flow diagram of this study (TIF 90 KB)

Supplementary file3 Supplemental Figure 3. The process of REPs surgery. (A) Surgical region was sterilized. (B) Distal and palatal root canals were sealed with resin. (C) The 15# K-file was inserted the root canal until the apical constriction. (D) No apparent bleeding was identified after 15# K-file was removed. (E) The canal was dried with 15 # paper points after irrigation. (F) No apparent bleeding was identified after drying the root canal. (G) New bleeding was induced up to the CEJ after breaking the apical constriction with new 15# K-file. (H) The root canal orifice and dental crown were covered with resin (TIF 377 KB)

## Data Availability

The analyzed data sets generated during the present study are available from the corresponding author on reasonable request.

## Abbreviations

REPs: Regenerative endodontic procedures
H&E: Hematoxylin and eosin
MT: Masson’s trichrome
μCT: Micro-computed tomography
MTA: Mineral trioxide aggregate
