# Supplementary figures and images for: Establishment of a regenerative endodontic procedures model of mature mouse teeth and evaluation of the wound healing process
Source: Odontology. 2025 Sep 29;114(3):1500–10. doi: 10.1007/s10266-025-01211-4 (PMC13319129; doi:10.1007/s10266-025-01211-4)

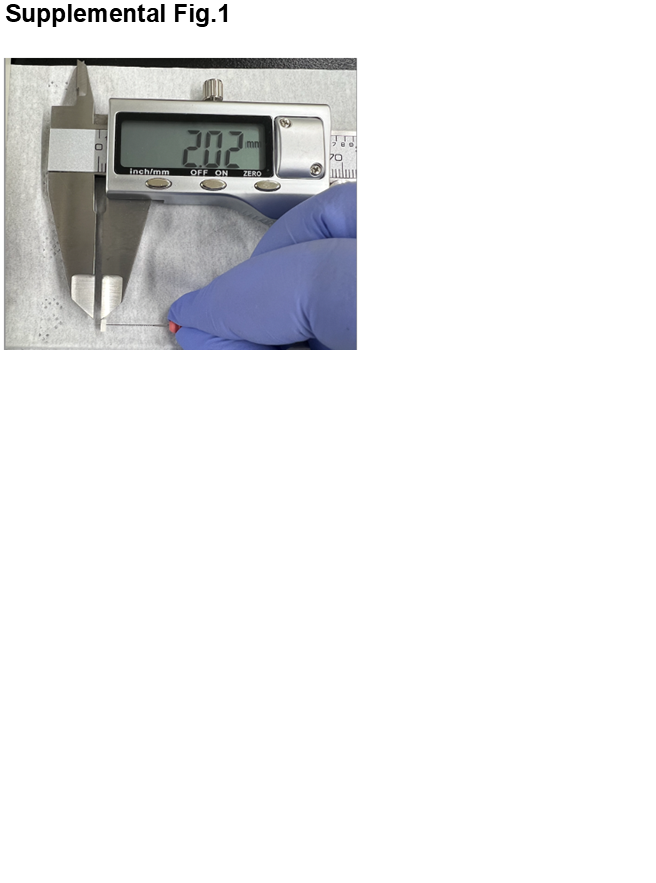

Supplement: Supplementary file 1 — Supplementary file1 Supplemental Figure 1. Working length measurement with electronic liquid crystal caliper (TIF 213 KB) [file 10266_2025_1211_MOESM1_ESM.tif]

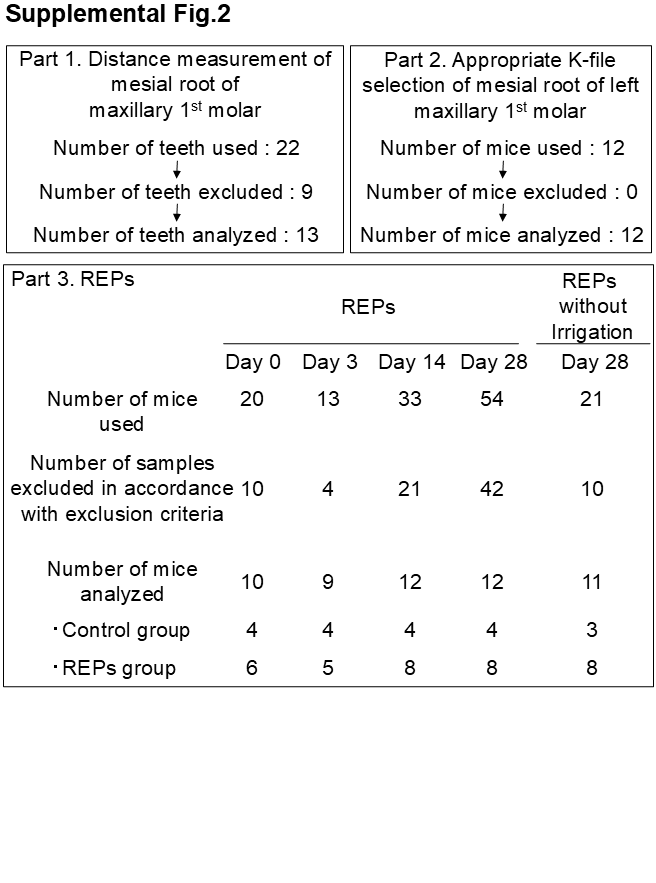

Supplement: Supplementary file 2 — Supplementary file2 Supplemental Figure 2. The flow diagram of this study (TIF 90 KB) [file 10266_2025_1211_MOESM2_ESM.tif]

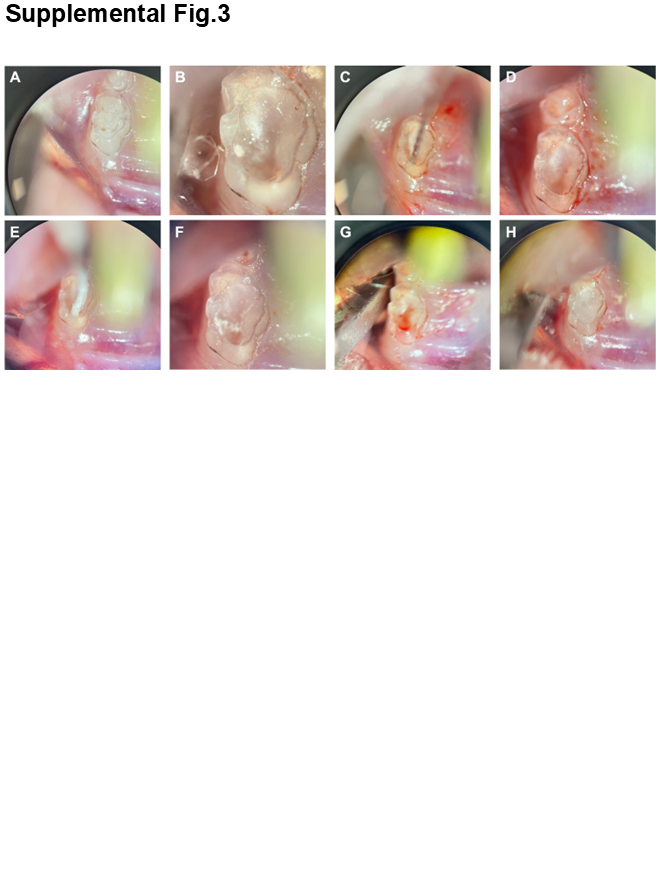

Supplement: Supplementary file 3 — Supplementary file3 Supplemental Figure 3. The process of REPs surgery. (A) Surgical region was sterilized. (B) Distal and palatal root canals were sealed with resin. (C) The 15# K-file was inserted the root canal until the apical constriction. (D) No apparent bleeding was identified after 15# K-file was removed. (E) The canal was dried with 15 # paper points after irrigation. (F) No apparent bleeding was identified after drying the root canal. (G) New bleeding was induced up to the CEJ after breaking the apical constriction with new 15# K-file. (H) The root canal orifice and dental crown were covered with resin (TIF 377 KB) [file 10266_2025_1211_MOESM3_ESM.tif]
